# Characteristics of adults involved in alcohol-related intimate partner violence: results from a nationally representative sample

**DOI:** 10.1186/1471-2458-14-466

**Published:** 2014-05-17

**Authors:** Jennifer M Reingle Gonzalez, Nadine M Connell, Michael S Businelle, Wesley G Jennings, Karen G Chartier

**Affiliations:** 1 School of Public Health, University of Texas Health Science Center at Houston, Dallas Regional Campus, 5323 Harry Hines Blvd, V8.112, Dallas, TX 75390, USA; 2 Program in Criminology, College of Economic, Political and Policy Sciences, The University of Texas at Dallas, 800 West Campbell Road, GR 31, Richardson, TX 75080, USA; 3 College of Behavioral and Community Sciences, Department of Criminology, University of South Florida, 4202 East Fowler Avenue, SOC 333, Tampa, FL 33620, USA; 4 School of Social Work, Virginia Commonwealth University, 1000 Floyd Ave, 3rd Floor, PO Box 842027, Richmond, VA 23284, USA

## Abstract

**Background:**

More than 12 million women and men are victims of partner violence each year. Although the health outcomes of partner violence have been well documented, we know very little about specific event-level characteristics that may provide implications for prevention and intervention of partner violence situations. Therefore, the purpose of this study is to evaluate substance abuse and dependence as risk factors for event-level alcohol-related intimate partner violence (IPV).

**Methods:**

Data were derived from Wave II of the National Epidemiological Survey on Alcohol and Related Conditions (2004–2005). Eligible participants (N = 2,255) reported IPV the year before the survey. Negative binomial and ordinal regression methods were used to assess risk factors for alcohol use during IPV.

**Results:**

Respondent PTSD was the only mental health diagnosis related to alcohol use during IPV (OR = 1.45). Marijuana use was related to respondents’ use of alcohol during IPV (OR = 2.68). Respondents’ meeting the criteria for alcohol abuse/dependence was strongly associated with respondent drinking (OR = 10.74) and partner drinking (OR = 2.89) during IPV.

**Conclusion:**

Results indicate that PTSD, marijuana use disorders, alcohol abuse and dependence are associated with more frequent alcohol use during IPV. In addition, it is important to consider that the patient who presents in emergency settings (e.g., hospitals or urgent care facilities) may not be immediately identifiable as the victim or the perpetrator of partner violence. Therefore, screening and intervention programs should probe to further assess the event-level characteristics of partner violence situations to ensure the correct service referrals are made to prevent partner violence.

## Background

Intimate partner violence (IPV) continues to be a substantial public health problem in the United States (U.S.), with an array of associated negative long-term health consequences, including depression, substance use, suicidality, sexually transmitted diseases, low self-esteem, and personal insecurity [1-8]. According to the National Intimate Partner and Sexual Violence Survey [9], more than 12 million women and men are victims of partner violence each year. During their lifetime, 35.6% of women and 28.5% of men in the United States have experienced some form of partner violence [9]. These numbers underscore the importance of better understanding the etiology of IPV and the role that various risk factors play in determining its prevalence and incidence. To date, however, limited understanding of the event-level correlates of IPV has hindered our ability to predict this behavior. Recent research points to the significance of the role that alcohol and drug use can have on IPV episodes, both in terms of long-term behavioral correlates and short-term escalating factors [10-12]. Specifically, alcohol use has been tied to IPV events as a potent risk factor for mutual aggression [13]. Therefore, prevention, screening and identification of alcohol use problems may emerge as a method of reducing IPV.

The association between alcohol and overall drug use and IPV is strong, with marijuana users and binge drinkers at increased risk for both being perpetrators and victims of IPV [12,14,15]. Given the evidence that alcohol and marijuana use often precedes IPV [12,16], research should focus on these risk factors at the event level. For instance, Caetano et al. [10] assessed event-level alcohol use, as well as psychosocial correlates, of IPV using a nationally representative sample. Although the focus of this study was on ethnic differences in alcohol use, Caetano and colleagues [10] reported that between 27 and 41% of men, and 4 to 24% of women, were drinking alcohol at the time of the violent event. Given these relatively large incidence rates, continued attention to the event-level relationship between substance use and IPV will advance our knowledge of how both distal and proximal factors related to substance use can influence IPV.

Research has also demonstrated the existence of persistent victim-offender overlap in IPV, with individuals acting as *both* a perpetrator and a victim during IPV events [11,12,17,18]. Indeed, research on the relationship between victims and offenders suggests that there are a substantial number of behavioral characteristics that predict both behaviors (see Schreck et al., [19] for a detailed overview). Given the potential for an IPV episode to escalate based on the behavior of one or more parties, it is important to examine this overlap (e.g., both victims and perpetrators) simultaneously, as these variables are not independent of one another.

The relationship between violence in general and alcohol use has been studied in detail, and four theories have been formulated to explain the relationship between alcohol use and violence, which directly apply to the role of alcohol use during IPV: (1) psychopharmacological, meaning that the intoxicating effects of alcohol cause people to be violent [20]; (2) the relationship is causal, in that alcohol use causes violence because aggressive people self-select into situations that encourage alcohol consumption [21]; (3) the relationship is reciprocal, and the arrow between alcohol use and violence may point in either or both directions [22]; or (4) the relationship is spurious, as problem behaviors cluster as part of a general problem behavior syndrome [23]. Furthermore, mental health is strongly related to alcohol use, drug use, and violent behavior; particularly, partner violence [16]. In this regard, alcohol and drug use may be self-medicating for the effects of IPV or other stressors; alternatively, they may be reducing self-control inhibitions and assisting in the perpetration and escalation of violence. Therefore, the current study will help clarify the relationship between event-level alcohol use and partner violence perpetration and victimization.

While previous studies have evaluated both substance-related and mental health risk factors for IPV [13,15,24], the current study will advance the literature by examining the correlates of alcohol use during IPV. In addition, this study will extend previous knowledge by examining the relation between *frequency* of alcohol use during IPV by both the respondent and their spouse/partner and perpetration and victimization. As such, the goals of this study are: 1) evaluate the frequency of alcohol use during IPV by both the respondent and their partner as a risk factor for perpetration and victimization of IPV; and 2) examine the mental health and substance use related correlates of alcohol consumption by each partner during IPV.

## Methods

Data were obtained from Wave II of the National Epidemiologic Survey on Alcohol and Related Conditions (NESARC) [25]. Respondents (*N* = 34,653) comprised a nationally representative sample of the U.S. adult population, including both citizens and non-citizens. Participants were eligible if they participated in Wave I (2001–2002) of NESARC (N = 43,093) and did not becoming ineligible (e.g., institutionalized, or no longer alive), resulting in an 81% response rate [25]. Wave I used a multistage stratified design and oversampled young adults (ages 18–24) and Non-Hispanic Black and Hispanic households. Data collection at Wave II occurred during 2004 and 2005. Sampling weights for Wave II consider the survey and sampling design characteristics of the NESARC survey as well as adjust for non-response and sample attrition, to maintain generalizability to the U.S. population. All secondary analyses were approved by the University of Texas Health Science Center at Houston Institutional Review Board.

In-person respondent interviews were administered by trained and experienced lay interviewers from the U.S. Census Bureau. The survey instrument was computerized with software that included built-in skip, logic, and consistency checks. Surveys were conducted either in English or Spanish based on respondent’s preference in face-to-face household settings. Supervisors re-contacted 10% of all respondents at random by telephone for quality control purposes.

For the purposes of the current study, respondents were included if they reported that they were "married, dating, or involved in a romantic relationship in the past year" *and* reported IPV in the past year (*n* = 2,255). Those who reported their relationship status within the prior year as "unknown" (n = 143) were excluded.

### Measures

#### Demographics

Respondents were asked to self-identify their age (retained as a continuous measure), sex (1 = male or 0 = female), and race/ethnicity at the time of the interview. Race/ethnicity was measured using the response options, "White, non-Hispanic", "Black, non-Hispanic", "American Indian/Alaska Native, non-Hispanic", "Asian/Native Hawaiian/Other Pacific Islander, non-Hispanic", and "Hispanic, any race". For analytical purposes, respondents were considered "White", "Black", "Other race" (due to small numbers of individual races), or "Hispanic".

#### Mental health disorders

All assessed mental health disorders (major depression, mania, dysthymia, hypomania, panic disorder, social phobia, specific phobia, general anxiety disorder, and post-traumatic stress disorder) in the past year were measured using the AUDADIS-IV (see [26]) and the DSM-IV-TR [27]. According to a psychometric analysis of the AUDADIS-IV [25,26,28], all indicators of mental health had particularly high internal consistency (α > 0.75), and these high rates of internal consistency were verified firsthand.

#### Substance use disorders

Marijuana use disorder in the past year was coded as "disordered" or "non-disordered/non-user" in accordance with the AUDADIS-IV [28]. Alcohol use disorders in the past year were also coded based on the AUDADIS-IV as "no alcohol abuse or dependence", "alcohol abuse only", and "alcohol dependence" (with or without alcohol abuse). Other drugs were not included due to a low sample size for individual drugs. We elected to focus on marijuana and alcohol specifically, rather than pooling "other drugs" into a diluted heterogeneous categorical variable, as marijuana has been related to IPV in previous research [12]. Previous studies have shown that alcohol and marijuana abuse and dependence, as measured in the AUDADIS-IV have Cronbach’s alpha coefficients exceeding 0.80 [26].

Each of these studies has documented that the AUDADIS-IV provides highly valid measures indicators of DSM criteria; however, they are much quicker and to administer and code in survey data [25,26,28].

*IPV*. Twelve items were used to create typologies of IPV victimization and perpetration. Specifically: 1) "How often did [you/your spouse or partner] push, grab, or shove [your spouse or partner/you] in the past year?"; 2) "How often did [you/your spouse or partner] slap, kick, bite or hit [your spouse or partner/you] in the past year?"; 3) "How often did [you/your spouse or partner] threaten [your spouse or partner/you] with a weapon like a knife or gun in the past year?"; 4) "How often did [you/your spouse or partner] cut or bruise [your spouse or partner/you] in the past year?"; 5) "How often did [you/your spouse or partner] force [your spouse or partner/you] to have sex in the past year?"; and 6) "How often did [you/your spouse or partner] injure [your spouse or partner/you] enough that [they/you] had to get medical care in the past year?". Response options for each of these items included, "Never", "once", "2-3 times", "once a month", and "more than once a month". These 12 items have been shown to have acceptable internal consistency for both participant reports (α = 0.70) and partner reports (α = 0.73), with acceptable reliability across samples (ICC_participant report_ = 0.79; ICC_partner report_ = 0.76) [28].

For the purposes of the current analyses, individuals were considered "exposed" to IPV if they reported that they were victims, perpetrators, or both, at least once within the past year. Using twelve IPV items, two dependent groups were created: 1) those who reported at least one victimization exposure; and 2) those who only reported perpetrating IPV at least once but no victimization exposure [11].

#### Event-level alcohol use

The following two questions were asked if respondents reported any IPV over the past 12 months: "How often had you been drinking at the time this/these [acts of intimate partner violence] happened?"; and "How often had your spouse or *partner been drinking at that time*?". Response options included, "never" (referent), "rarely", "some of the time", and "always" for both the respondent and their spouse.

### Analytical methods

Consistent with the recommendations for NESARC data analysis, all analyses were conducted considering the clustered two-stage sampling design, and observations were weighted due to the unequal probability of selection of each primary sampling unit [29]. Survey χ^2^ analyses, negative binomial and ordinal regression were performed to address the specific aims of this study.

Two models were used to characterize the substance- and mental health-related comorbidities of alcohol use during IPV. First, we tested the hypothesis that the frequency of drinking by each partner during IPV was related to the victimization and/or perpetration of IPV by the respondent using a bivariate probit model. This type of model was utilized because the two dependent variables (IPV perpetration and victimization) are intrinsically correlated. Therefore, both models were modeled simultaneously. We then evaluated the bivariate relationships between alcohol abuse and dependence, substance use, mental health, and demographic correlates and perpetration and victimization of IPV using an ordinal regression framework. Variables that were related to alcohol use during IPV (bivariate relation *p* < .10) for either perpetration or victimization were included in the main effects multivariate models. All statistical analyses were conducted using the STATA 12 analytical package (StataCorp, College Station, TX).

## Results

The sample (N = 2,255), comprised of those who reported some form of IPV in the past year, was 45.8% male, 58.6% White or Caucasian, 17.7% Black or African-American, and 16.1% Hispanic or Latino. The average age of respondents was 40.1 (SE = 0.42). Seventy-five percent of the sample reported victimization from IPV, and 74% reported IPV perpetration (49% reported both perpetration and victimization; therefore, the percentages exceed 100%). Demographic characteristics, as well as self-reported substance use and mental health disorders, are detailed in Table 1.

**Table 1:** Sample description of those who reported intimate partner violence in the past year, NESARC II, n = 2,255

	**N**	**%**
Intimate Partner Violence (IPV, Total N) in past year*		
Victimization	1,760	75.14
Perpetration	1,824	73.97
*Demographics*		
Male	919	45.77
Race and Ethnicity		
White or Caucasian	1,088	58.61
Black or African-American	647	17.71
Hispanic or Latino	549	16.13
Other race	124	7.55
Age^a^	40.08 (0.42)	
*Risk Factors (past year)*		
Marijuana use disorder	130	5.70
Alcohol abuse & dependence		
No alcohol abuse or dependence	1,930	79.15
Alcohol abuse or dependence	478	19.95
Depression	452	17.60
Mania	186	7.41
Dysthymia	72	2.43
Hypomania	85	3.66
Panic disorder	103	4.36
Social phobia	142	5.76
Specific phobia	322	13.04
General Anxiety	219	8.99
Post-Traumatic Stress Disorder	377	14.39
*Event-level alcohol use: Respondent (n = 1,675)*		
None	1,423	84.43
Rarely	166	9.60
Sometimes	30	1.92
Always	56	4.06
*Event-level alcohol use: Spouse/partner (n = 1.608)*		
None	1,120	71.07
Rarely	255	15.37
Sometimes	92	4.99
Always	141	8.57

^a^Mean and SE are reported.

*Victimization and perpetration exceed 100%, as 49% of the sample has been both victims and perpetrators of IPV.

In most cases, alcohol was never used during IPV (71.1% for spouse using alcohol; 84.4% for respondent using alcohol). Four percent of respondents reported that they always use alcohol during IPV; while 8.6% of respondents reported that their spouses always use alcohol during IPV.

As detailed in Table 2, the frequency of alcohol use during IPV by the respondent was closely related to the likelihood of victimization, but this relationship did not hold for perpetration. Similarly, reports of partner drinking during IPV were also related to respondent victimization, but not perpetration of IPV.

**Table 2:** Bivariate probit regression analysis of event-level frequency of alcohol use as a risk factor for perpetration and victimization during IPV

	***b***	***95% CI***	***b***	***95% CI***
*Respondent drinking during IPV, n = 1504*				
None (referent)	Ref	--	Ref	--
Rarely	.60***	.34-.86	.45***	.30-.60
Sometimes	.46	-.34-1.27	.19	-.09-.47
Always	.29	-.76-1.34	-.02	-.38-.35
*Respondents’ spouse drinking during IPV, n* = *1565*				
None (referent)	Ref	--	Ref	--
Rarely	.33***	.20-.47	.34**	.09-.60
Sometimes	.53***	.35-.71	-.01	-.31-.29
Always	.36*	.36-.68	-.07	-.37-.22

Note. Ref = Reference group that was omitted for comparison.

*p < 0.05.

**p < 0.01.

***p < 0.001.

There were significant bivariate differences in alcohol abuse and dependence, and marijuana use disorders across the alcohol use groups (i.e., respondent drinking or spouse drinking) during IPV (Table 3). Presence of mental health disorders (e.g., depression, generalized anxiety disorder, panic disorder) was not related to drinking during IPV episodes with the exception of PTSD, which was associated with reported partner drinking (OR = 1.74; 95% CI 1.22-2.48). Based upon these results, further analyses focused specifically on marijuana, alcohol abuse/dependence, and PTSD as risk factors for alcohol use during IPV.

**Table 3:** Ordinal regression analysis examining the relationship between demographics, alcohol and marijuana use disorders and alcohol use during IPV by the respondent

	**Respondent drinking**		**Spouse drinking**	
	**OR**	**95% CI**	**OR**	**95% CI**
*Respondent Demographics*				
Male	1.95**	1.29-2.95	.64**	.46-.89
Race and Ethnicity				
White or Caucasian	Ref	--	Ref	--
Black or African-American	.82	.50-1.34	.68*	.50-.93
Hispanic or Latino	.96	.53-1.73	.80	.52-1.21
Other race	1.20	.50-2.89	.55	.19-1.60
Age	.99	.97-1.00	.99	.98-1.01
*Risk Factors (Respondent; past year)*				
Marijuana use disorder	5.59***	2.95-10.60	2.31**	1.46-3.68
Alcohol abuse or dependence	12.17***	7.08-20.94	2.75***	1.78-4.25
Depression	1.50	.84-2.68	1.48	.93-2.36
Mania	1.98	.87-4.52	1.17	.62-2.18
Dysthymia	.85	.35-2.05	1.09	.49-2.43
Hypomania	1.29	.46-3.58	1.90	.77-4.70
Panic disorder	1.58	.95-2.63	1.80	.84-3.89
Social phobia	.79	.32-1.94	.70	.34-1.42
Specific phobia	.87	.53-1.42	1.22	.79-1.90
General Anxiety	.83	.42-1.63	.84	.48-1.47
Post-Traumatic Stress Disorder	1.25	.66-2.36	1.74**	1.22-2.48

Note. Ref = Reference group that was omitted for comparison.

*p < 0.05.

**p < 0.01.

***p < 0.001.

Table 4 depicts the results of ordinal survey multivariate regression analyses that examined correlates between a marijuana use, alcohol abuse/dependence, demographics, and the ordinal outcome of alcohol use during IPV, stratified by the reported alcohol user. Results indicated that the respondents who had a marijuana use disorder were more likely to report respondent drinking alcohol during IPV (OR = 2.68; 95% CI 1.36-5.25) compared to respondents without a marijuana use disorder. Presence of alcohol abuse and dependence was strongly related to an increased likelihood of respondent drinking (OR = 10.74; 95% CI 6.41-17.99) and partner drinking (OR = 2.89; 95% CI 1.77-4.75) during IPV episodes. Respondents who have been diagnosed with PTSD in the previous year were more likely than non-diagnosed respondents to report that their spouse or partner drinks alcohol during IPV events (OR = 1.45; 95% CI 1.01-2.09). Male respondents were less likely to report drinking by their spouse (OR = .53; 95% CI .37-.77) during IPV events; however, there were no gender differences in respondent drinking during IPV. African-Americans were less likely than Whites to report spouse or partner drinking (OR = .68; 95% CI .50-.91).

**Table 4:** Multivariate ordinal regression analysis examining the relationship between demographics, alcohol and marijuana use disorders and alcohol use during IPV by the respondent

	**Respondent drinking**		**Spouse drinking**	
	**OR**	**95% CI**	**OR**	**95% CI**
*Respondent Demographics*				
Male	1.30	.77-2.20	.53**	.37-.77
Race and Ethnicity				
White or Caucasian	Ref	--	Ref	--
Black or African-American	.73	.42-1.26	.68*	.50-.91
Hispanic or Latino	.91	.46-1.78	.90	.58-1.39
Other race	1.23	.46-3.25	.63	.23-1.73
Age	1.01	.99-1.02	1.01	.99-1.02
*Risk Factors (Respondent; past year)*				
Marijuana use disorder	2.68**	1.36-5.25	1.67	.92-3.01
Alcohol abuse or dependence	10.74***	6.41-17.99	2.89***	1.77-4.75
Post-Traumatic Stress Disorder	1.09	.54-2.21	1.45*	1.01-2.09

Note. Ref = Reference group that was omitted for comparison.

*p < 0.05.

**p < 0.01.

***p < 0.001.

## Discussion

In summary, results from this study suggest that alcohol abuse and dependence, as well as marijuana use, are the most robust correlates of alcohol use during IPV. Mental health disorders, with the exception of PTSD, were not significantly related to alcohol use IPV. These inform our understanding of the correlates of alcohol use during IPV events. Alcohol use during IPV is an important prevention target, as use of alcohol during violent incidents has been theorized to increase impulsive behavior and may escalate violent events [20]. The correlates identified in this paper (PTSD, alcohol abuse/dependence, marijuana use) may be identified by a physician as indicators of the initiation of IPV behavior in a relationship, providing a clear context for screening and brief intervention. This is an important direction to be pursued in future research.

These findings add to the previous research, which has indicated that alcohol and marijuana use generally precede the IPV event [12,16,24], and we were able to expand the literature base by including PTSD as a risk factor for alcohol use during IPV. This may be due to differences in measurement of alcohol and marijuana use, as much of the research on IPV has had limited event-level measures. Respondents who reported alcohol abuse and dependence were significantly more likely than those without alcohol abuse or dependence to report that themselves and their partners consume alcohol during IPV. This finding indicates that alcohol use may be a particular harbinger for IPV events among those with a recent alcohol use disorder. Alcohol is known to reduce inhibitions toward violence, and it appears that this may be particularly true in those with alcohol use disorders.

The finding that mental health disorders (e.g., depression, anxiety) were not correlated with alcohol use during IPV perpetration or victimization was particularly interesting. This result appears to indicate that alcohol use is the primary risk factor for IPV, which is often attributed to mental health. It was not surprising that the respondents’ recent diagnosis of PTSD is related to partner drinking, as IPV victimization has been identified as a risk factor for PTSD in international studies [30].

These results should be considered in light of several limitations. First, these data are cross-sectional in nature and data on alcohol use were obtained from only one partner, which may result in some inaccurate reporting of bi-directional IPV, as well as alcohol use by the spouse or partner during IPV [10]. Furthermore, the quantity of alcohol consumed by each partner during an IPV event is unknown, and quantity of alcohol consumed could be associated with aggressive behavior. Unfortunately, the data did not capture the length of time each partner spent drinking, or how temporally proximal their alcohol consumption was to the IPV event. Finally, the alcohol-related IPV measures that were used may be subject to social desirability biases and associated under-reporting of alcohol use during IPV. Nevertheless, this study is the first exploration of risk factors for alcohol-related IPV (considering both substance use and mental health disorders simultaneously while modeling the dependency between victimization and perpetration of IPV), including groups of adults who are both perpetrators and victims, using a nationally representative sample of adults in the United States.

The findings of the current study have important policy implications. For example, prevention and intervention efforts targeting IPV should take into account that in any IPV episode, the roles of victim and perpetrator may not necessarily be clearly discernible (as approximately half of those participating in IPV are both victims and perpetrators). In this same vein, these types of incidents could involve parties who have been victims and/or perpetrators previously and their role in any particular incident may be more complex than measured at that particular point in time. Therefore, the individual who presents in an emergency setting (e.g., a hospitals or urgent care facility) may not be immediately identifiable (or validly identifiable) as the victim or the perpetrator. Therefore, screening and intervention programs should probe to further assess the event-level characteristics of partner violence situations. It is also important that victim services and perpetrator interventions take into account the possibility that these may not be mutually exclusive categories and some individuals may have experienced victimization or have perpetrated IPV as well. Also, the results indicate that prevention and intervention strategies should target modifiable risk factors, particularly alcohol abuse and dependence, as well as marijuana use disorders and PTSD, in order to identify, prevent, and reduce the occurrence and reoccurrence of IPV.

## Conclusion

In conclusion, this research adds to the literature by classifying the frequency of alcohol use during IPV instances specific to those who are victims and perpetrators of IPV. Furthermore, we found support for alcohol abuse and dependence, PTSD, and marijuana use disorders to be strongly correlated with alcohol use during IPV. This finding lays the foundation for future research that will investigate the relationship between event-level alcohol use and victimization and perpetration of IPV, ideally quantity and frequency reported by both partners). Future research should refine the measurement of alcohol-related IPV to evaluate event-specific instances of partner violence, and the perceived role in which alcohol use directly impacts IPV event severity.

## Competing interests

The authors declare that they have no competing interests.

## Authors’ contributions

JMRG conceptualized the study, conducted all data analysis, and wrote the results section. NMC drafted the introduction and thoroughly revised all sections of the manuscript. MSB provided substantial conceptual feedback and revised the draft into its current form. WGJ drafted the discussion and implications sections. KC provided the data and drafted the methodology section. All authors have read and approved the final manuscript.

## Pre-publication history

The pre-publication history for this paper can be accessed here:

http://www.biomedcentral.com/1471-2458/14/466/prepub
